# Disseminated peritoneal leiomyomatosis post-laparoscopic myomectomy

**DOI:** 10.1093/bjrcr/uaag039

**Published:** 2026-08-26

**Authors:** Dana AlNuaimi, Shareefa Abdulghaffar, Reem AlKetbi, Badr Ahmed

**Affiliations:** Department of Health, Abu Dhabi 20224, United Arab Emirates; Dubai Health, Dubai 1853, United Arab Emirates; Dubai Health, Dubai 1853, United Arab Emirates; Dubai Health, Dubai 1853, United Arab Emirates

**Keywords:** uterine fibroids, leiomyomas, disseminated peritoneal leiomyomas, laparoscopic morcellation, myomectomy

## Abstract

Disseminated peritoneal leiomyomatosis (DPL) is a rare benign condition in which scattered smooth muscle nodules resembling leiomyomas grow in multiple extrauterine sites within the peritoneal cavity, mimicking malignant processes like peritoneal carcinomatosis. The etiology of this condition is unclear, but it is thought to be due to either iatrogenic or hormonal factors, as studies have found that DPL is more common in females of reproductive age and in those who have undergone laparoscopic surgery with morcellation of uterine leiomyomas, as seen in the present case. Diagnostic imaging plays an important role in the preoperative diagnosis and in accurately delineating the extent of the disease. Nevertheless, histopathology remains the gold standard for a definitive diagnosis. We report a case of a 38-year-old female patient with a prior history of laparoscopic myomectomy who presented 2 years later with nonspecific left flank pain. Contrast-enhanced CT of the abdomen and pelvis and MRI revealed multiple enhancing nodular lesions scattered within the abdominal cavity. Given the prior history of laparoscopic myomectomy and radiological findings, DPL was suspected, which was confirmed later by histopathological examination.

## Introduction

Leiomyomas are the most common benign uterine tumors, composed of smooth muscle cells, and they often occur in females of reproductive age, with prevalence rates as high as 20%-30%.^1^^,^^2^ Leiomyomas may rarely occur in unusual extra-uterine locations such as the peritoneal cavity or retroperitoneal spaces and may be disseminated; thus, they are termed disseminated peritoneal leiomyomatosis (DPL), retroperitoneal leiomyomatosis, and parasitic leiomyomas.^2^

Disseminated peritoneal leiomyomatosis is an extremely rare condition characterized by smooth-muscle tumors that grow along the peritoneal surfaces within the abdominopelvic cavity, with less than 200 cases reported in the literature.^1–3^ The pathogenesis is still unclear, but it is hypothesized that hormonal and iatrogenic factors play a role, especially in patients post-myomectomy with morcellation, as seen in the present case.^3^

Despite its benign nature, there is a minute risk of malignant transformation into leiomyosarcoma. In addition, multiple tumors may lead to a misdiagnosis of metastatic disease; therefore, medical imaging plays a crucial role in establishing a provisional diagnosis, especially when correlated with the patient’s relevant clinical and surgical history.^4^

## Case report

A 38-year-old female patient presented to the emergency department complaining of left flank pain. There was no associated nausea or vomiting, nor were there any urinary symptoms. The patient’s past medical history included a laparoscopic myomectomy 2 years prior. No other significant medical history was reported.

On physical examination, the patient’s vitals were stable. Chest, abdominal, and neurological examinations were normal.

The patient’s laboratory blood work-up, including full blood count, electrolytes, liver and kidney function tests was unremarkable. Pancreatic enzymes were not elevated. urine analysis was normal. Tumor markers were negative.

An abdominal x-ray showed no radioopaque renal calculi or dilated bowel loops.

**Figure 1:**
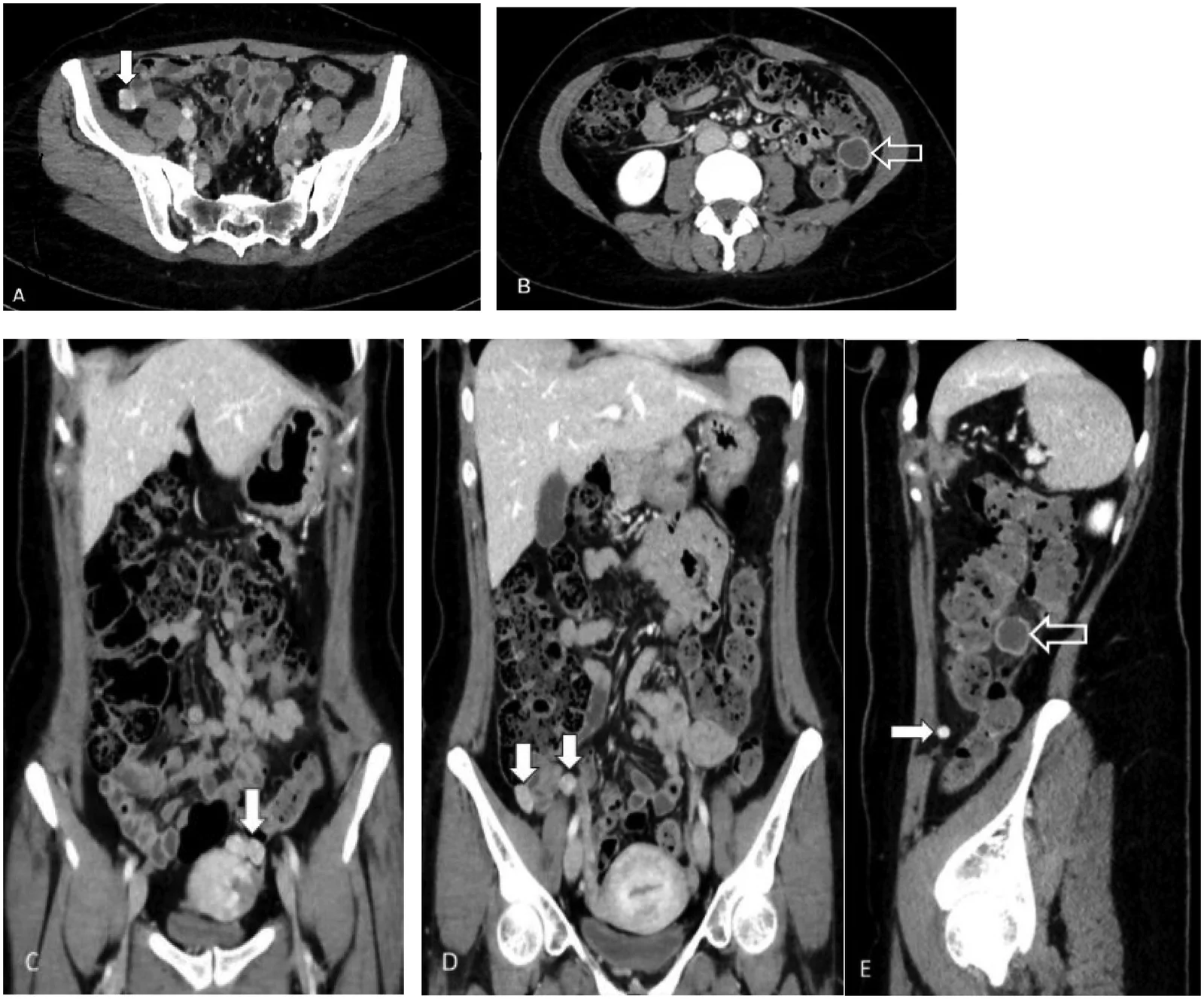
Enhanced CT of the abdomen and pelvis at the portovenous phase: (A) Axial: A solid peritoneal nodule is seen in the right iliac fossa adjacent to distal ileal loops. (B) Axial: A cystic nodule demonstrating fluid attenuation in HU values, with a thin enhancing rim is located at the left lateral aspect of the peritoneal cavity, insinuated between the mid-segment of the descending colon and the distal segment of the transverse colon. (C) Coronal: A solid homogeneously enhancing nodule is seen at the left superolateral aspect of the uterine operative bed. (D) Coronal: Two homogeneously enhancing solid nodules are located medial and lateral to distal ileal loops. (E) Sagittal: A cystic nodule (empty arrow) and a tiny solid peritoneal nodule (filled arrow).

A contrast-enhanced CT scan of the abdomen and pelvis was performed, which revealed multiple scattered small enhancing nodular lesions at the operative bed and within the peritoneal cavity. The largest of these appeared cystic, measured 2 × 2 cm in diameter, and was located adjacent to the mid-segment of the descending colon and showed peripheral rim enhancement with surrounding subtle fat stranding (Figure 1).

Otherwise, the solid and hollow abdominal organs were unremarkable. There was no evidence of ascites, retroperitoneal or pelvic lymphadenopathy.

The provisional diagnosis of a disseminated peritoneal process was made, and the patient was admitted for further work-up and surgical planning.

A gadolinium-enhanced MRI of the abdomen and pelvis showed a normal-sized anteverted uterus with an operative scar at the left anterolateral aspect of the uterine fundus, corresponding to the site of previous myomectomy.

**Figure 2:**
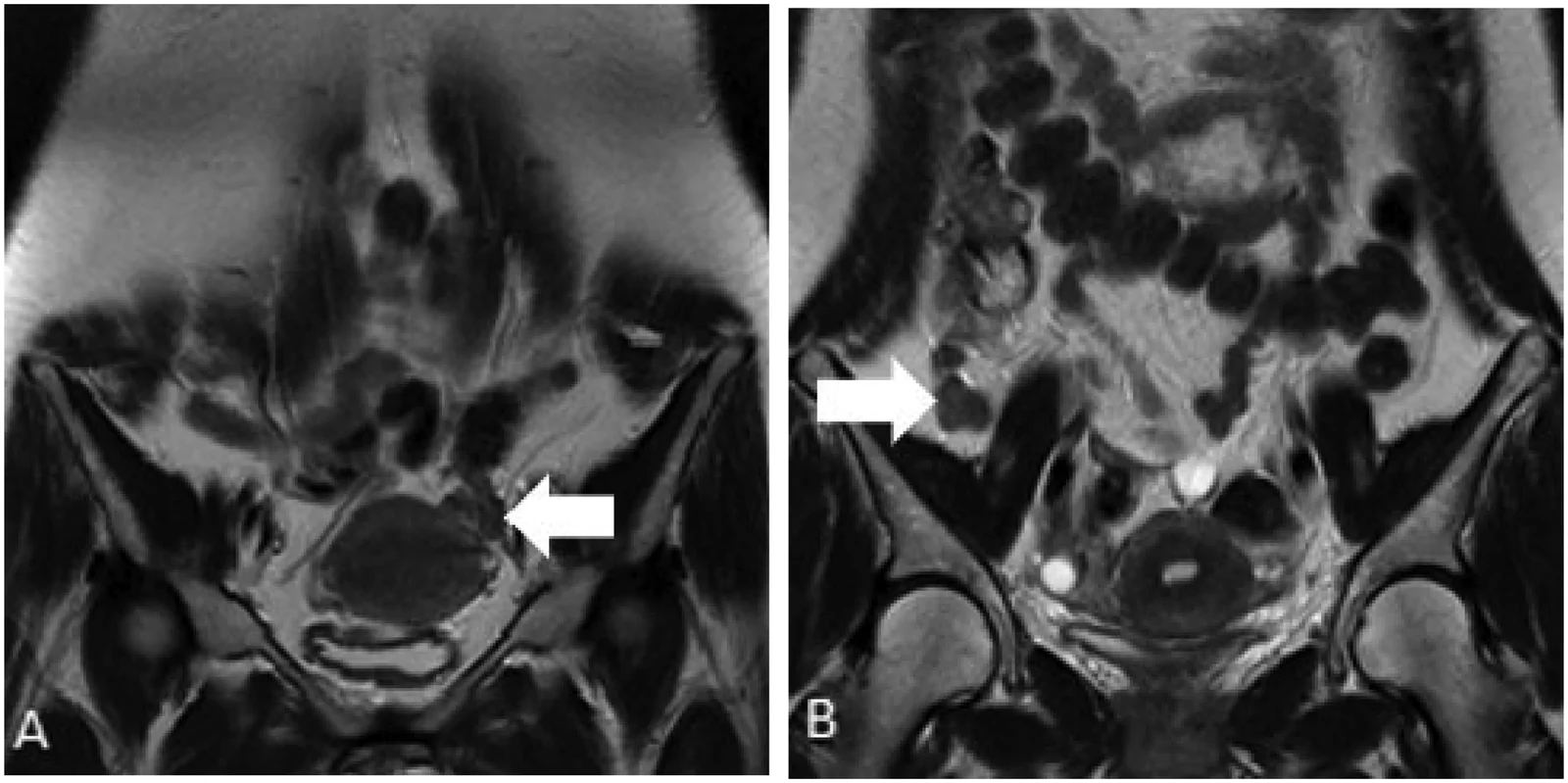
MRI of the abdomen and pelvis in coronal T2WI showing a similar signal pattern to that of the solid pedunculated uterine/operative bed leiomyoma (A) and a nodular peritoneal lesion adjacent to the right distal ileal loop (B).

The solid peritoneal nodular lesions visualized on the CT images were also noted on MRI, exhibiting hypointense signal on T1WI and T2WI, mild post-contrast enhancement, and no evidence of diffusion restriction (Figure 2).

**Figure 3:**
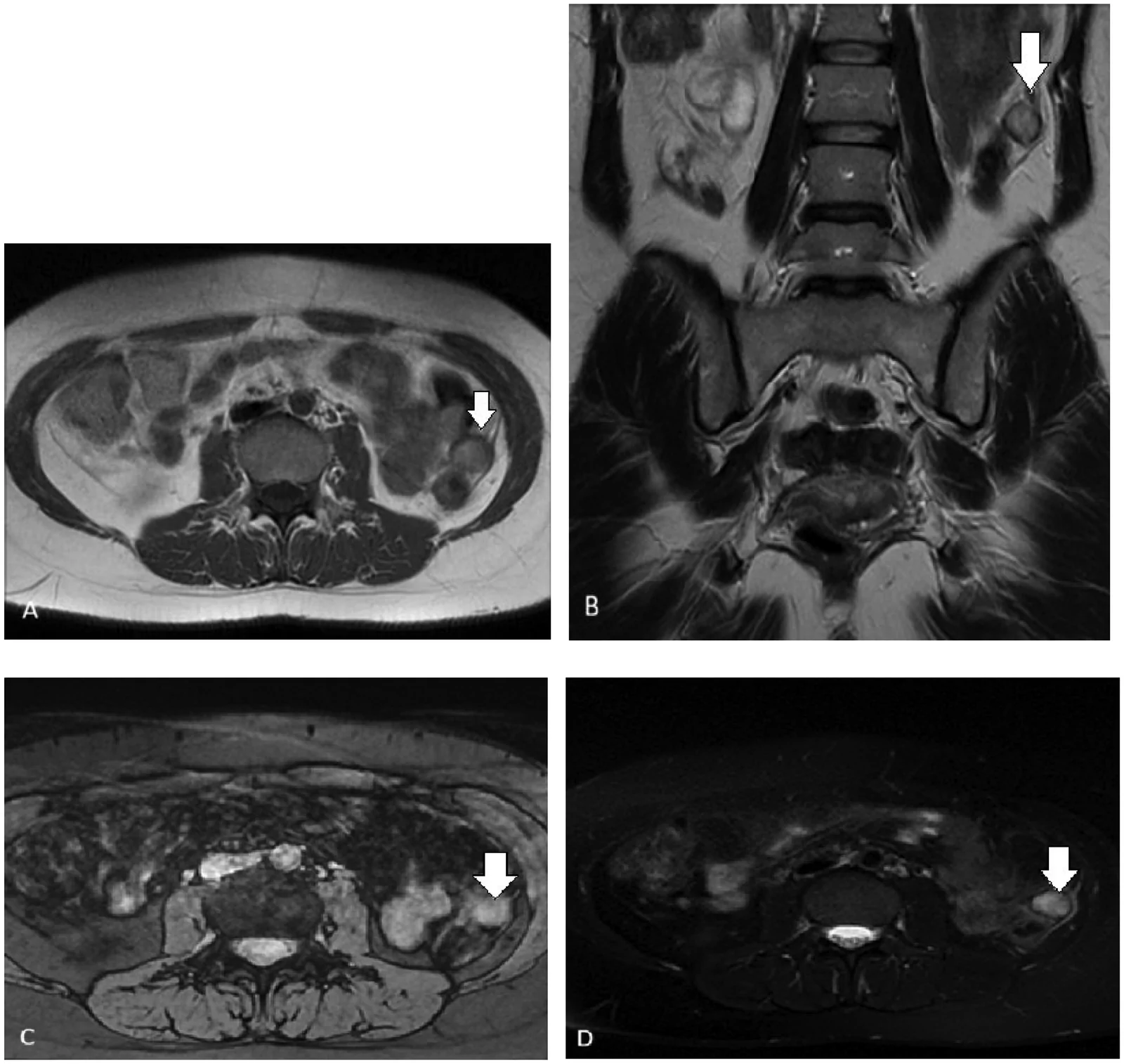
MRI of the abdomen and pelvis. (A) Axial T1WI and (B) Coronal T2WI showing a cystic nodule adjacent to the descending colon, exhibiting intermediate to hyperintense signal on both sequences. (C) Axial T2* GRE WI showing no blooming artifacts. (D) Axial T2FS WI revealing a persistent hyperintense signal on the fat suppression sequence.

The cystic peritoneal nodule demonstrated an intermediate to hyperintense signal on T1WI and T2WI. No blooming artifacts were noted on the GRE sequence to suggest hemorrhagic components. No fat components were detected on fat suppression sequences (Figure 3).

Review of the previous operative notes revealed that the patient had undergone a laparoscopic myomectomy with morcellation of a large fundal uterine leiomyoma, which measured 11 × 9 cm in diameter. Considering the previous history of myomectomy and the recurrent small uterine leiomyoma at the operative site, along with multiple peritoneal nodular implants with a similar signal intensity pattern to the coexisting uterine leiomyoma, a diagnosis of disseminated peritoneal leiomyomas was highly suggested. The cystic nodule seen close to the sigmoid colon likely represents cystic degeneration of the leiomyoma, which may explain the patient’s current presentation.

Other differential diagnoses, including peritoneal endometriotic deposits and carcinomatosis, were considered less likely, as there was no evidence of endometriosis, ascites, or lymphadenopathy on imaging.

**Figure 4:**
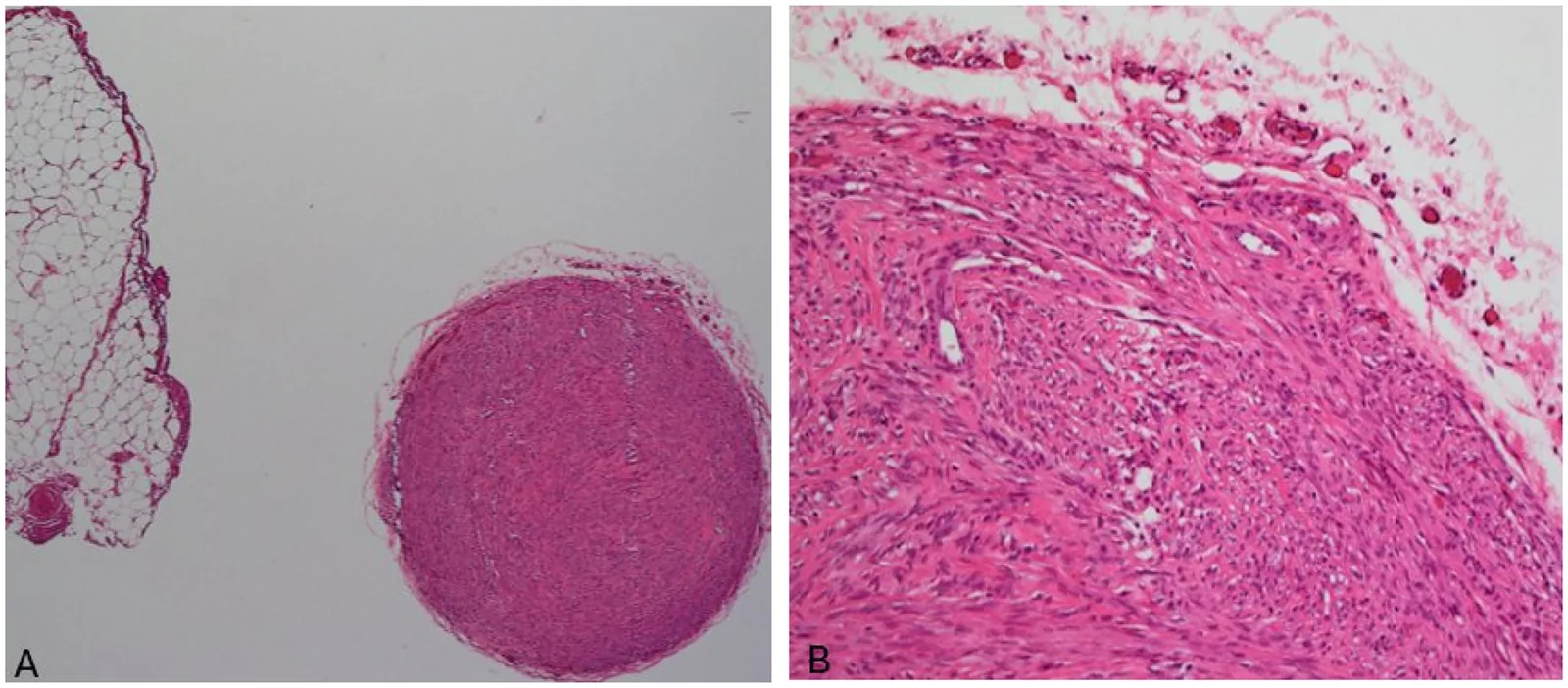
Histopathology images of the excised soft tissue nodules. (A) Hematoxylin and eosin stain, ×40, showing a round, solid nodule of whorled, intersecting bundles of smooth muscle spindle cells adjacent to omental fatty tissue. (B) Hematoxylin and eosin stain, ×200, of the peritoneal nodule showing intersecting eosinophilic smooth muscle spindle cell fascicles with the periphery showing congested peritoneal tissue.

The patient underwent surgical laparoscopy to remove the nodular peritoneal lesions. Histopathology of the excised lesions was consistent with the diagnosis of multiple leiomyomas, showing the conventional spindle cell type with no evidence of atypia, mitosis, or necrosis (Figure 4).

The patient had an uncomplicated postoperative course and was discharged home with a follow-up appointment at the OBGYN clinic in 6 weeks.

## Discussion

Disseminated peritoneal leiomyomatosis is a rare benign condition characterized by the implantation of multiple discrete smooth muscle nodules that resemble uterine leiomyomas macroscopically and histologically along the peritoneal and omental surfaces.^1^

The pathogenesis of this condition is unknown; however, proposed theories have included hormonal factors, in which excess endogenous or exogenous estrogen levels can stimulate smooth muscle cell differentiation within the peritoneum, supported by the fact that DPL is more common in women within the childbearing period, in those on oral contraceptives, during pregnancy, and in women with ovarian tumors secreting gonadal hormones.^4^^,^^5^ Another theory is iatrogenic, as DPL has been more commonly seen in patients who have undergone morcellation of large myomas during laparoscopy leading to peritoneal seeding along the trocar sites, with a reported incidence rate of 0.95%.^3^^,^^4^

Patients may be asymptomatic or present with nonspecific symptoms such as abdominal discomfort or pelvic pain. In asymptomatic patients, the lesions may be discovered incidentally during medical imaging procedures or abdominal surgery.^1–3^ Symptoms depend mainly on the site of disease, as nodular involvement of the peritoneum along the bowel loops can lead to bowel obstruction, and nodules arising near the bladder can lead to urinary symptoms.^1^^,^^3^

Medical imaging plays a crucial role in the provisional diagnosis, preoperative planning to determine the extent of the disease, and assessing the treatment response.^5^ CT depicts multiple isodense soft tissue nodules of homogeneous or heterogeneous attenuation and variable enhancement patterns.^2^ MRI usually shows multiple peritoneal soft tissue nodules with low signal intensity on both T1 and T2-weighted images, similar to smooth muscle and contrast enhancement of the solid component post-contrast administration. PET may be used in selected cases; peritoneal leiomyomatosis typically demonstrates absent or low-grade FDG uptake, reflecting its benign smooth muscle origin, whereas peritoneal carcinomatosis more commonly shows increased metabolic activity.^5^

Peritoneal carcinomatosis is an important differential diagnosis when multiple peritoneal nodules are seen on imaging; however, it is an advanced disease process that presents with other associated findings such as peritoneal thickening, omental caking, adenopathy, and ascites with an identifiable primary malignancy.^3^^,^^5^^,^^6^

Other differential diagnoses of DPL include endometriosis, splenosis, peritoneal mesothelioma, lymphoma, and the fibrotic type of peritoneal tuberculosis.

Endometriosis findings are nonspecific on CT, and the endometriotic nodules may appear solid, cystic, or mixed. MRI is more sensitive for detecting endometriotic nodules, demonstrating fibrotic nodular or retractile masses of low signal intensity on T2WI, with or without hyperintense hemorrhagic foci on T1WI with fat suppression.^6^

Splenosis is usually discovered incidentally on imaging and may be solitary or multiple splenic implants, routinely measuring less than 3 cm in diameter. They exhibit attenuation values identical to normal splenic tissue on both CT and MRI. Scintigraphic studies are the gold standard diagnostic modality and are highly sensitive and specific for splenic uptake using 99mTc heat-damaged erythrocytes or Indium 111-labeled platelets.^6^

Peritoneal mesothelioma is commonly seen in middle-aged men and manifests as peritoneal thickening with associated extensive calcifications.^1^^,^^2^

Lymphoma involving the peritoneum is also characterized by peritoneal thickening with predominant lymphadenopathy, ascites, mesenteric, and solid organ involvement.^6^

The fibrotic type of peritoneal tuberculosis usually reveals hypoattenuating nodules associated with necrotic mesenteric lymphadenopathy.^2^

Histopathology provides the definitive diagnosis of DPL and confirms the presence or absence of malignant transformation. Disseminated peritoneal leiomyomatosis appears in histopathology as fusiform smooth muscle cells, occasionally containing fibroblasts and myofibroblasts, without atypia and in a morphological arrangement similar to leiomyomas.^7^

There is no standard treatment protocol for DPL, and both medical and surgical treatment options may be tailored to accordance with the patient’s case, and depending on their age, family planning options, and disease complications.^4^^,^^7^^,^^8^

## Conclusion

Disseminated peritoneal leiomyomatosis is a rare benign disease with nonspecific clinical symptoms, and patients are frequently misdiagnosed with other peritoneal neoplastic diseases. A high index of suspicion is necessary to establish a provisional diagnosis in female patients of childbearing age with a prior history of laparoscopic myomectomy where morcellation was used, as seen in the present case. Although DPL is a benign condition, malignant transformation has been reported, and close surveillance is recommended. Diagnostic imaging plays a crucial role in establishing the preoperative provisional diagnosis among the various differential diagnoses and in delineating the extent of the disease. Radiological assessment, particularly cross-sectional studies, provides valuable information regarding the number, size, distribution, attenuation values, and signal characteristics of the lesions, as well as assessing their relationship to adjacent abdominal and pelvic structures. Imaging findings may support the diagnosis of peritoneal leiomyomatosis while aiding in differentiating it from other conditions, particularly peritoneal carcinomatosis, thereby facilitating appropriate surgical planning.

## Learning points

- Disseminated peritoneal leiomyomatosis is a rare benign condition in which smooth muscle leiomyomas grow in multiple extrauterine sites within the peritoneal cavity. A high index of suspicion is necessary for diagnosis in female patients of childbearing age with a history of laparoscopic myomectomy where morcellation was used.
- Medical imaging plays an important role in the preoperative provisional diagnosis and in delineating the extent of the disease regarding the number, size, distribution, attenuation values, and signal characteristics of the lesions, as well as assessing their relationship to adjacent abdominal and pelvic structures.
- Radiological findings may support the diagnosis of peritoneal leiomyomatosis while aiding in differentiating it from other conditions, particularly peritoneal carcinomatosis, thereby facilitating appropriate surgical planning. However, histopathology remains the gold standard for a definitive diagnosis.
